# Age- and sex-based evaluation of the association between refractive error and age-related macular degeneration in the Korean population

**DOI:** 10.1371/journal.pone.0228468

**Published:** 2020-01-29

**Authors:** Kook Lee, Jin-Woo Kwon, Wan Jin Jahng, Young-Hoon Park, Donghyun Jee

**Affiliations:** 1 Department of Ophthalmology and Visual Science, Seoul St. Mary’s Hospital, College of Medicine, The Catholic University of Korea, Seoul, Korea; 2 Department of Ophthalmology and Visual Science, St. Vincent’s Hospital, College of Medicine, The Catholic University of Korea, Suwon, Korea; 3 Department of Petroleum Chemistry, American University of Nigeria, Yola, Nigeria; 4 Catholic Institute for Visual Science, College of Medicine, The Catholic University of Korea, Seoul, Korea; University Hospitals Cleveland, UNITED STATES

## Abstract

**Purpose:**

The aim of this study was to investigate the association between refractive error and prevalence of age-related macular degeneration (AMD) in Korean adults, based on the sex and age group.

**Methods:**

This was a nationwide population-based cross-sectional study that included 17,676 subjects aged over 40 years who participated in the 2008–2012 Korean National Health and Nutrition Examination Survey. Digital fundus images (45°) were obtained for both eyes under physiologic mydriasis and were graded using the international classification and grading system for age-related macular degeneration. The spherical equivalents of refractive errors were calculated in diopters using auto-refraction data.

**Results:**

After adjustment for potential confounders, myopia was associated with lower risk of any age-related macular degeneration [odds ratio (OR), 0.74; 95% Confidence Interval (CI), 0.61–0.91]. In particular, myopia was significantly associated with lower odds of age-related macular degeneration in female participants (any AMD: OR, 0.71; 95% CI, 0.54–0.93; early AMD: OR, 0.70; 95% CI, 0.53–0.93) and in participants younger than 50 years (any AMD: OR, 0.46; 95% CI, 0.24–0.90; early AMD: OR, 0.47; 95% CI, 0.24–0.93). There was no significant association between myopia and age-related macular degeneration in male participants and in participants older than 50 years.

**Conclusions:**

In the Korean adult population, myopia was associated with significantly lower odds of any type of early age-related macular degeneration, particularly in females and in younger age groups.

## Introduction

Age-related macular degeneration (AMD) is a major cause of blindness in people older than 60 years.[1–3] Several genetic and environmental factors such as aging, smoking history, and a family history of AMD have been recognized as risk factors for the development and exacerbation of AMD.[4, 5] Other potential factors including cardiovascular disease, dietary oxidant intake, and sunlight exposure have been inconsistently associated with the development of AMD.[6–9] We previously reported that age, sex, male, and hypertension were risk factors for AMD in a representative Korean population.[10]

Myopia is a common refractive error affecting approximately 1.6 billion people globally. The prevalence of myopia has increased over several decades at an epidemic rate, especially in East Asia.[11] Recent studies have suggested that refractive error may be associated with AMD. Some studies reported a lower risk of AMD in myopic eyes, whereas others failed to discover/establish such a relationship.[12–15] A recent study on multi-ethnic Asian cohorts suggested that reduced risk of AMD in myopes was observed in Chinese men.[16]

In our previous studies, we reported that the prevalence of early- and late-AMD was 6.0% and 0.6%, respectively, in a Korean population aged over 40 years, which was similar to or higher than the percentages recorded in other Asian countries.[10] In a follow-up study to identify the causative factors of AMD, sex-related differences were found in the correlation of vitamin D, cadmium, and lead with AMD.[17–19] In addition, we reported that the prevalence of myopia in Korean adults over 40 years was 34.7% and that there is a tendency of rapid increase in myopia among Korean adults.[20] As both AMD and myopia are important ocular conditions affecting Korean adults, and are associated with severe visual impairment, it is imperative to analyze the relationship between AMD and myopia in the Korean population.

In this study, we investigated the relationship between refractive error and AMD in Korean adults using representative population-based data from the Korean National Health and Nutrition Examination Survey (KNHANES). Particularly, we analyzed in greater detail whether the association between AMD and myopia varies according to the sex and age group.

## Methods

This study used data acquired from KNHANES. KNHANES is a nationwide and population-based cross-sectional study. The survey consists of a health interview, a nutritional survey, and a health examination survey. The KNHANES adopted a rolling sampling design, which is a stratified, complex, multistage, probability cluster survey with proportional allocation based on the National Census Registry for the non-institutional civilian population of Korea. Details about the study design and methods have been reported previously.[21, 22]

**Fig 1:**
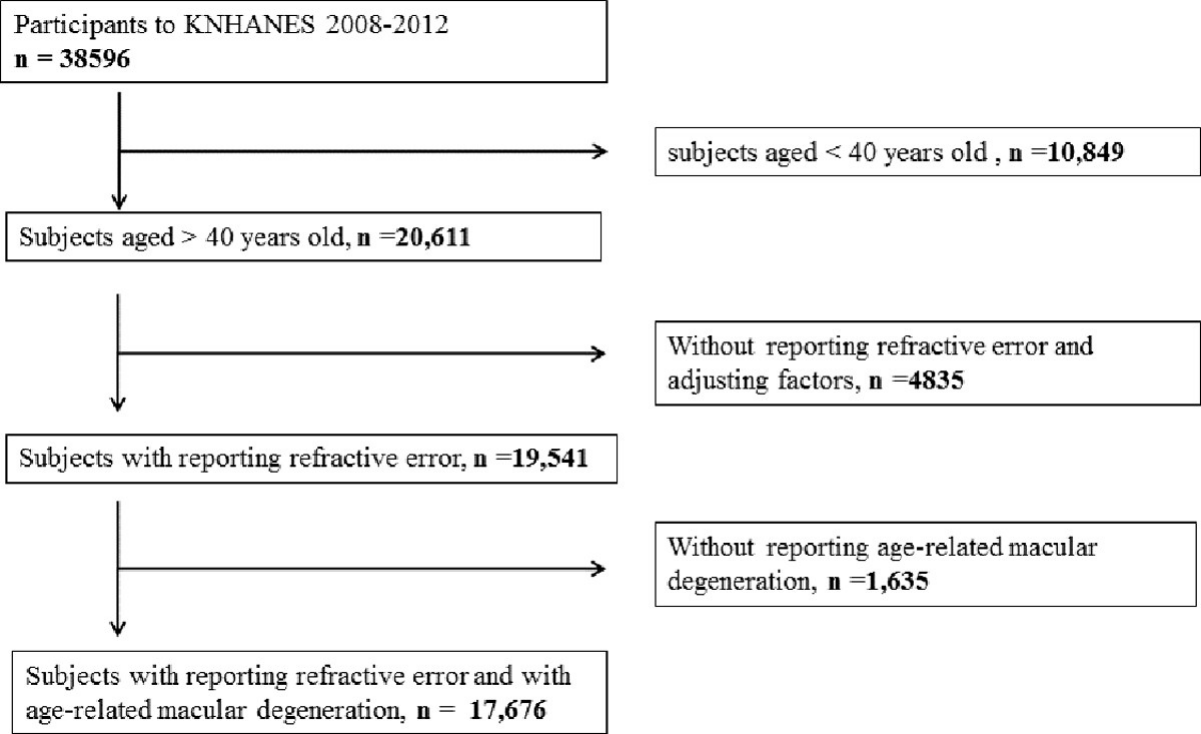
Flow diagram showing selection of study participants.

We included data obtained from KNHANES 2008–2012. Of the 38,596 individuals who took part in KNHANES, 10,849 participants aged under 40 years old, 4,835 participants without refractive error data, and 1,635 participants who did not undergo fundus examinations were excluded from the present study. Thus, 17,676 participants were included in the final analysis (Fig 1). All participants gave written informed consent. This study design followed the tenets of the Declaration of Helsinki for biomedical research and was approved by the Institutional Review Board of the Catholic University of Korea in Seoul, Korea.

Digital fundus images were obtained under physiological mydriasis using a digital fundus camera (TRC-NW6S, Topcon, Tokyo, Japan). For each participant, a 45° digital retinal image, centered on the fovea, was obtained for each eye (two images per person in total). Each image was graded twice using the international classification and grading system for AMD.[22, 23] Two different graders analyzed the fundus photographs, and a trained senior grader who is an ophthalmologist, was consulted in case of disagreements. The graders were masked to the patients' characteristics and entrusted by the Korean Ophthalmologic Society. The inter-rater reliability for AMD grading between the preliminary and detailed grading in the right and left eyes was 90.2% and 90.7% in 2008, 92.4% and 93.3% in 2009, 94.1% and 95.0% in 2010, 96.2% and 96.6% in 2011, and 96.0% and 96.2% in 2012. The quality of the survey was verified by the Epidemiologic Survey Committee of the Korean Ophthalmologic Society. Training of participating resident doctors was performed periodically by acting staff members of the National Epidemiologic Survey Committee of the Korean Ophthalmologic Society.

Early-AMD was defined by the presence of soft, indistinct, or reticular drusen on the macula, any type of drusen plus hyper- or hypo-pigmentary changes to the retinal pigment epithelium (RPE) in the macula, or by the presence of soft drusen without late-AMD signs on the macula. Late-AMD was defined by the presence of one of the following lesions: detachment of the RPE or neurosensory retina, hemorrhages in the subretinal or sub-RPE space, disciform scar, or geographic atrophy as a discrete depigmented area with visible choroidal vessels.[24, 25] For subjects with AMD lesions in only one eye, or asymmetric AMD lesions in both eyes, the worse eye was considered. Subjects with typical findings associated with pathologic myopia such as posterior staphyloma, lacquer crack, and patchy atrophy were not considered as AMD to distinguish from the myopic degeneration.[26]

Refractive error was measured using an auto-refractor (KR-8800®; Topcon, Tokyo, Japan) without cycloplegia. Average values of three refraction measurements were printed out from the auto-refractor. The spherical equivalent (SE) of refractive error was calculated as the spherical value plus half of the astigmatic value. Low myopia was defined as SE less than ^−^0.50 diopters (D) and high myopia was defined as SE less than 6.0 D.

Demographic and social factor data were based on the information from health interview. The presence of diabetes mellitus was defined as a fasting blood glucose level of greater than or equal to 126 mg/dL or subjects on antiglycemic medication. The presence of hypertension was defined as systolic blood pressure greater than or equal to 140 mm Hg and diastolic blood pressure greater than or equal to 90 mm Hg, or subjects on antihypertensive medication. Data on current sunlight exposure time were obtained from two possible answers to a single question: less than five hours or greater than or equal to five hours per day. Smoking status was determined with a self-reporting method that required a choice from two possible options: current smoker and never smoker. Educational level of subjects was classified into four categories: elementary school graduate, middle school graduate, high school graduate, and college graduate and above. Economic status was grouped into quartiles according to annual individual earnings.

The SPSS® version 18.0 (SPSS, Chicago, IL, USA) was used for statistical analyses. Since KNHANES used a stratified, multistage sampling method, we incorporated sampling weights as well as strata for the sampled units in the statistical analysis. Continuous variables were presented as mean and standard error (SE), and categorical variables were presented as percentage and SE. To compare the patients’ demographic characteristics, one-way analysis of variance (ANOVA) or chi-square tests were used. To evaluate the association of myopia and AMD, multiple logistic regression analyses were performed. After calculation of the crude odds ratio, these values were adjusted for confounders that were established as risk factors in previous studies, including age, sex, smoking, hypertension, and sunlight exposure time.[8, 10, 27] Adjusted odds ratios for those without myopia (>-0.5 D) and with myopia (<-0.5 D) were calculated for each stratum of sex. We also performed separate regression models stratified by sex. All variables for logistic regression analysis were examined for multicollinearity, and only variables with a variance inflation factor less than 10 were used. P values were two-tailed, and P less than 0.05 indicated statistical significance.

## Results

A total of 17,676 subjects aged over 40 years with available data on refractive error and fundus photographs were eligible for the study. The demographic characteristics of the 17,676 enrolled subjects by AMD status are summarized in Table 1. Subjects with AMD were more likely to be older (P < 0.001), hypertensive (P < 0.001), have longer sun exposure (P < 0.001), smokers (P < 0.001), and were less likely to be myopia (P < 0.001) than those without AMD.

**Table 1:** Demographic and clinical characteristics, according to early- and late- age-related macular degeneration (AMD) status and participation status, as reported in the Korean National Health and Nutrition Examination Survey 2008–2012.

Characteristics	No AMD (n = 16,308)	Early-AMD (n = 1,249)	Late-AMD (n = 123)	*P*	Participants (n = 17,676)
**Male (%)**	48.3 (0.4)	40.8 (1.7)	62.6 (5.4)	< .001*	47.9 (0.4)
**Age (yrs)**	54.3 (0.1)	64.9 (0.3)	65.9 (1.3)	< .001*	61.6 (0.4)
**Diabetes (%)**	12.3 (0.4)	15.0 (1.3)	7.2 (2.6)	.020*	12.4 (0.3)
**Hypertension (%)**	37.6 (0.6)	52.5 (1.8)	56.9 (5.6)	< .001*	38.6 (0.5)
**Sun exposure (>5hrs/day, %)**	20.0 (0.7)	26.1 (1.8)	23.7 (5.0)	< .001*	20.4 (0.7)
**Smoking status**				< .001*	
Never (%)	54.3 (0.4)	60.7 (1.8)	37.9 (5.5)		54.6 (0.4)
Former(%)	15.7 (0.4)	15.0 (1.3)	22.6 (5.3)		15.7 (0.4)
Current (%)	30.0 (0.5)	24.3 (1.5)	39.5 (5.6)		29.8 (0.4)
**Income levels**				.799	
1st quartile (lowest)	25.7 (0.6)	25.1 (1.6)	26.7 (4.9)		25.7 (0.6)
2nd quartile	26.1 (0.5)	25.9(1.6)	20.4(4.4)		26.1(0.5)
3rd quartile	24.4 (0.5)	26.3(1.6)	25.5(4.8)		24.6 (0.5)
4th quartile	23.7 (0.6)	22.7 (1.5)	27.4 (5.2)		23.7 (0.6)
**Education levels**				< .001*	
1st quartile (lowest)	28.9 (0.6)	55.0(1.8)	45.8 (5.5)		30.5 (0.6)
2nd quartile	15.8 (0.4)	17.2 (1.4)	15.2 (4.3)		15.8 (0.4)
3rd quartile	33.7 (0.6)	18.2 (1.3)	25.4 (5.1)		32.8 (0.5)
4th quartile	21.6 (0.7)	9.6 (1.1)	13.6 (4.3)		20.9 (0.6)
**Refractive Error (D)**				< .001*	
**Myopia (%)**	42.5 (0.5)	25.3 (1.7)	29.5 (5.2)		41.5 (0.5)

Datas are expressed as weighted means or weighted frequency (%) with standard errors.

P values compared patients with any AMD and without AMD

*p < 0.05

Table 2 shows odds ratios (ORs) for the association of myopia and AMD status based on sex and age. The adjusted ORs for any and early AMD were 0.74 (95% CI, 0.61–0.91, p = 0.004) and 0.74 (95% CI, 0.59–0.91, p = 0.006) respectively. In females, the adjusted ORs for any and early AMD were 0.71 (95% CI, 0.54–0.93, p = 0.015) and 0.70 (95% CI, 0.53–0.93, p = 0.014) respectively. There was no significant association between myopia and any or early AMD in male participants. Among participants younger than 50 years, the adjusted ORs for any and early AMD were 0.46 (95% CI, 0.24–0.90, p = 0.024) and 0.47 (95% CI, 0.24–0.93, p = 0.030), respectively. In contrast, there was no significant correlation between myopia and any or early AMD in participants older than 50 years. There was no significant association between late AMD and myopia.

**Table 2:** Association between myopia and any AMD, early AMD, and late AMD.

Myopia	Ors	95% CI	*P*
**Any AMD**			
Total	0.74	0.61–0.91	.004*
Men	0.76	0.56–1.03	.081
Women	0.71	0.54–0.93	.015*
<50 years	0.46	0.24–0.90	.024*
>50 years	0.85	0.69–1.04	.127
**Early AMD**			
Total	0.74	0.59–0.91	.006*
Men	0.76	0.56–1.05	.103
Women	0.70	0.53–0.93	.014*
<50 years	0.47	0.24–0.93	.030*
>50 years	0.84	0.67–1.05	.131
**Late AMD**			
Total	0.81	0.46–1.44	.487
Men	0.69	0.30–1.59	.390
Women	0.94	0.42–2.12	.893
<50 years	0.25	0.02–2.47	.240
>50 years	0.95	0.52–1.74	.877

Odds Ratio was expressed with 95% confidence intervals after adjusted for sex, age, diabetes, hypertension, smoking, income levels, education levels.

* p < 0.05 Prevalence was expressed as weighted estimates [%] (standard errors [%], 95% confidence intervals).

Table 3 shows ORs for association of myopia and any AMD according to age groups. There was a significant association between myopia and AMD in younger age groups (< 50 years) (ORs, 0.46; 95% CI, 0.24-.90 in 40–49 years). However, there was no significant association between any AMD and myopia in older age groups (older than 50 years).

**Table 3:** Association between myopia and any AMD according to age group.

Myopia	Ors	95% CI	*P*
**Any AMD**			
Total	0.74	0.61–0.91	.004
40–49 years	0.46	0.24–0.90	.024
50–59 years	0.97	0.66–1.41	.877
60–69 years	0.76	0.54–1.08	.136
> 70 years	0.89	0.67–1.19	.460

Odds Ratio was expressed with 95% confidence intervals after adjusted for sex, age, diabetes, hypertension, smoking, income levels, education levels.

* p < 0.05 Prevalence was expressed as weighted estimates [%] (standard errors [%], 95% confidence intervals).

Table 4 shows the change of ORs of refractive error for any AMD after adjusting for potential covariates such as age, sex, hypertension, smoking, diabetes, sunlight exposure, income levels, and education levels. The association between refractive error and any AMD showed positive correlation even after adjusting for potential confounders. The odds ratio decreased by only 0.1 from 1.24 to 1.14, even though the analysis was performed by adding adjusting variables.

**Table 4:** Change of Odds ratio (OR) of refractive error (Diopter) for any AMD in multiple logistic regression in Korean. Odds ratios were expressed with 95% confidence intervals (CI).

Model	Ors	95% CI	*P*
AMD = RE + constant	1.60	1.52–1.69	< .001
AMD = RE + age + constant	1.24	1.08–1.43	< .001
AMD = RE + age + sex +constant	1.13	1.09–1.18	< .001
AMD = RE + age + sex + hypertension + constant	1.13	1.09–1.18	< .001
AMD = RE + age + sex + hypertension + smoking + constant	1.13	1.08–1.18	< .001
AMD = RE + age + sex + hypertension + smoking + diabetes + constant	1.15	1.09–1.20	< .001
AMD = RE + age + sex + hypertension + smoking + diabetes + sunlight exposure + constant	1.15	1.10–1.20	< .001
AMD = RE + age + sex + hypertension + smoking + diabetes + sunlight exposure + income + constant	1.15	1.09–1.20	< .001
AMD = RE + age + sex + hypertension + smoking + diabetes + sunlight exposure + income + education + constant	1.14	1.08–1.19	< .001

RE, refractive error; CI, Confidence interval

Table 5 shows the change of ORs of myopia for and AMD after adjusting for potential covariates listed above. The adjusted ORs significantly showed negative correlation even after adjusting for potential confounders.

**Table 5:** Change of Odds ratio (OR) of myopia (≤ -0.5diopter [D]) for any AMD in multiple logistic regression in Korean. Odds ratios were expressed with 95% confidence intervals (CI).

Model	Ors	95% CI	*P*
AMD = myopia + constant	0.46	0.39–0.55	< .001
AMD = myopia + sex + constant	0.46	0.39–0.55	< .001
AMD = myopia + sex + age +constant	0.73	0.62–0.87	.001
AMD = myopia + sex + age + hypertension + constant	0.75	0.63–0.89	.002
AMD = myopia + sex + age + hypertension + smoking + constant	0.75	0.63–0.90	.002
AMD = myopia + sex + age + hypertension + smoking + diabetes + constant	0.72	0.59–0.88	.001
AMD = myopia + sex + age + hypertension + smoking + diabetes + sunlight exposure + constant	0.72	0.60–0.88	.002
AMD = myopia + sex + age + hypertension + smoking + diabetes + sunlight exposure + income + constant	0.72	0.59–0.88	.002
AMD = myopia + sex + age + hypertension + smoking + diabetes + sunlight exposure + income + education + constant	0.74	0.61–0.91	.004

CI, Confidence interval

Using a Wald test for coefficient of interaction term to evaluate effect modification, we found a statistically significant interaction based on sex between myopia and any AMD (P for interaction < 0.001), between myopia and early AMD (P for interaction <0.001), and between myopia and late AMD (P for interaction = 0.006) (Table 6).

**Table 6:** Effect modification between myopia and age-related macular degeneration (AMD) by gender among representative Korean adults aged 40 years or older included in the study. Association was expressed as odds ratio (OR) with 95% confidence intervals.

	Without myopia (> -0.5D)		With myopia (< -0.5D)		P for interaction
	OR	95% CI	OR	95% CI	
**Any AMD**					
Men	1.00	reference	0.47	0.37, 0.61	
Women	1.24	1.07, 1.45	0.57	0.46, 0.72	< .001
**Early AMD**					
Men	1.00	reference	0.46	0.35–0.60	
Women	1.36	1.16–1.59	0.62	0.49–0.80	< .001
**Late AMD**					
Men	1.00	reference	0.57	0.30–1.09	
Women	0.52	0.30–0.89	0.30	0.14–0.64	.006

## Discussion

The present study showed that the association between myopia and AMD was particularly significant in females and younger age groups (< 50 years) but not in males and older age groups (> 50 years). In addition, these associations were revealed to be independent factors that did not change even after adjusting for various confounding factors.

AMD is a disease well-known to be the leading cause of irreversible blindness and severe visual loss in older populations in both European and Asian countries. Previously, several studies have suggested that refractive error may be associated with AMD, also among both European and Asian populations. A population-based survey of Australia showed a statistically significant increased risk of early AMD for each diopter of increase in refractive error (OR: 1.1; CI: 1.0–1.2).[28] The Rotterdam Study, a population-based cohort study in Netherlands, revealed that the age- and sex-adjusted OR of AMD prevalence for every diopter of progress toward hyperopia was 1.09 (CI: 1.04–1.13).[12] For Asian population-based studies, Cheung et al reported that myopia (< -0.5 D) was significantly associated with a lower risk for AMD (OR, 0.44) in Chinese men.[16] In 2012, a study conducted in India showed that each diopter of increase in hyperopia was associated with 15% (OR: 1.15; CI: 1.06, 1.24] increased probability of early AMD.[13] The Singapore Indian Eye Study also found a negative association between myopia and AMD (OR, 0.45; CI, 0.25–0.79).[29] A recently published study based on the Korean population showed that each diopter of increase in refractive error was associated with an increased risk of any and early AMD (OR: 1.16, 1.18, respectively)[30]; this result is consistent with the results of the present study.

Although the reasons for the association between refractive error and AMD remain unclear, several possible explanations for them have been proposed over the past few decades. One hypothesis is based on the difference in scleral rigidity between myopic and hyperopic eyes. Previous studies[31, 32] have found that hyperopic eyes are more likely to have increased scleral rigidity, which leads to impairment of the transfer of nutrients and oxygen to the outer retina. Increased scleral rigidity may affect the choroidal function such as thermoregulation to protect the retina from damage in extreme hot or cold temperature and it could result in oxidative stress and retinal damage caused by reactive oxygen intermediates, which can contribute to the pathogenesis of AMD.[33, 34] Pallikaris et al also reported that patients with neovascular AMD had more increased ocular rigidity than non-neovascular AMD and control patients.[31] Myopic eyes with longer axial length are known to have a less rigid and compact sclera compared with hyperopic eyes;[32] these differences in structural features may explain the relationship between refractive error and the prevalence and distribution of AMD based on refractive error. Another hypothesis is the decreased intraocular concentration of vascular endothelial growth factor (VEGF) in myopic eyes. It is thought that the intraocular concentration of VEGF decreases significantly with increasing myopia and increasing axial length,[35] which can result in decreased angiogenesis. The use of spectacles by myopic patients is also a possible reason for this association because of the attendant reduction in exposure to ultraviolet rays in sunlight, a known risk factor for AMD.[36, 37] The other hypothesis is the application of Laplace’s law. According to this law, vascular tension increases as intravascular pressure increases or vascular radius increases. It is known that there is reduced blood flow in high myopic eyes, due to narrowing of the retinal vessel diameter.[38, 39] Other studies have also reported decreased density and capillary diameter in the choriocapillary layer of myopic eyes using an animal model and human data.[40, 41] Therefore, it can be inferred that the decreased vascular diameter in myopia leads to reduction of vascular tension, which may lead to reduced occurrence of AMD. Recent published study which performed Medelian randomization analysis also provided genetic evidence that there is a causal relationship between refractive error and AMD, although the causal effect size was modest (OR, 1.08).[42]

Unlike previously published studies on the Korean population,[30] our study further analyzed whether the relationship between refractive error and AMD is affected by sex or age. First, the analysis of our results according to sex showed that there was a statistically significant negative correlation of myopia with any AMD (OR, 0.71; CI, 0.54–0.93) and early AMD (OR, 0.70; CI, 0.53–0.93) in the female group; however, these relationships were not statistically significant in the male group. The exact explanation for these differences according to sex has not been clarified yet. We considered that this could be due to the hormonal differences between males and females. Based on a few studies, it is known that the sex and hormonal status could influence choroidal circulation. Kavroulaki et al found that there was significantly reduced choroidal blood flow in postmenopausal women compared to premenopausal women,[43], and Centofanti et al suggested that estrogen may be responsible for these changes.[44] In the present study, we only included participants older than 40 years; therefore, it is estimated that majority of the enrolled women may be in the postmenopausal stage, although no information on their menopause status was provided. Thus, it can be assumed that unlike in males, changes in choroidal blood flow due to hormonal changes may have affected AMD development in females; this was manifested in our results as a sex-related difference in AMD prevalence. Further detailed investigations are needed to support this explanation.

According to the analysis based on age group, our results showed that negative correlation between myopia and AMD was more significant in the younger age group (< 50 years) than in the older (> 50 years) age group. This association between myopia and AMD was only significant in the participants in their 40s, but not in those in their 50s, 60s, or 70s. The reason for the significant result in the total analysis is that the strength of the association between myopia and AMD in the 40s age group is quite large. Thus, it is suggested that myopia at young age has an inverse association with the occurrence of AMD. These age-related differences could be described from the aspect of posterior vitreous detachment (PVD). The prevalence of PVD significantly increases with aging.[45–47] In addition, myopic eyes are more likely to have PVD.[48, 49] It has been demonstrated that the frequency of PVD is lower in patients with exudative AMD than in controls because persistent vitreomacular adhesion might induce chronic low-grade inflammation, prevent normal oxygen and nutrient diffusion to the macula, and/or confine proangiogenic cytokines in the macula.[50–52] Some researchers suggest that PVD protects against exudative AMD.[53]. The prevalence of PVD may be more common in myopic eyes than in non-myopic eyes among younger participants. Thus, it can be suggested that young myopic subjects with PVD may be less likely to have AMD.

The main strength of the present study is the relatively large number of participants (n = 17,676) and the study design of systemic, stratified, multistage, clustered, random sampling methods. Another strength of our study is that we investigated the differences in sex- and age-specific associations between myopia and the prevalence of AMD, which has not been reported in previous studies. However, our study had some limitations. Firstly, refractive errors were evaluated without cycloplegia, which may lead to overestimation of myopia. Secondly, several factors associated with refractive status, such as axial length and corneal curvature, were not available in the population-based data used in the present study. However, to overcome these limitations, our researchers are in the process of analyzing axial length data in a population of more than 10,000 subjects collated from the KNHANES database. Thirdly, there was no statistically significant association between refractive error and late AMD, probably due to the small number of subjects with late AMD in this population. Fourthly, this study has the risk of clumping AMD and myopic degeneration together. Because the fundus finding of myopic degeneration is similar to that of AMD, some of myopic degeneration might be interpreted as AMD. However, the prevalence of AMD was lower in myopia than in non-myopia in KNHANES database. Although the results of our study may have overestimated the prevalence of AMD in myopia, the errors inherently are most likely to reduce the chance of finding associations, so the association found in the present study may even be stronger than detected. The confounding that myopic degeneration may be interpreted as AMD would be negative confounding which may cause underestimate of true association. Thus, this bias could support our conclusion. Finally, our study had a cross-sectional design, which makes inferring causality difficult. However, we documented associations between refractive status and AMD on the basis of existing evidences regarding refractive change and AMD development.

In conclusion, the present study provides population-based epidemiological data on the association between refractive error and AMD in a representative Korean population. We found that myopia was associated with significantly lower odds of any or early AMD after adjusting for potential confounders; this association was particularly prominent in females or in the younger age group. In the future, further well-designed longitudinal studies are required to confirm the association between refractive error and AMD, and to clarify the mechanism underlying this association.
